# Biogeotechnology, Biocorrosion, and Remediation—Three Areas of Modern Applied Environmental Microbiology

**DOI:** 10.3390/microorganisms10081611

**Published:** 2022-08-09

**Authors:** Mikhail Vainshtein, Tatiana Abashina

**Affiliations:** Federal Research Center “Pushchino Scientific Center for Biological Research of the Russian Academy of Sciences”, Skryabin Institute of Biochemistry and Physiology of Microorganisms RAS, Prospect Nauki 5, 142290 Pushchino, Russia

For many years, medical microbiology and food microbiology have been the most studied areas of microbial biology. This is an understandable situation, since they provide knowledge that primarily determines the survival of people. Moreover, even when people did not have a clear understanding of microorganisms, they were already introducing rules of thumb for microbiological control.

Microbiological technologies for the rational use of the environment, as well as the fight against corrosion and toxic pollution, appeared less than 100 years ago. With the development of industry and the reduction of natural resources, these areas of microbiology are becoming increasingly important for all mankind. The rapid development of industrial technology requires the optimization of the use of natural resources and secondary resources, as well as the neutralization of an increasing variety of toxicants.

Chemical processing methods of hydrometallurgy are highly effective, but this approach requires the introduction of a large amount of active reagents into the environment. Thus, processing requires further improvement, since it is not only a source of valuable metals, but also can lead to the formation of acidic mine effluents. In turn, these effluents provoke a large-scale technogenic disaster, i.e., the acidification of mining areas with strong inorganic acids [1,2].

Microorganisms are inherent catalysts for many chemical reactions that do not occur spontaneously in nature or require a large consumption of reagents. In addition, there are pollutants with a specific chemical composition that take an extremely long time to decompose without microorganisms. Taken together, these factors stimulated the development of new biogeotechnologies and, at the same time, some scientific progress in modern applied microbiology of the environment in general.

The first steps in this direction were carried out in the biogeotechnology of leaching, namely, in leaching gold [3]. Since the 1950s, chemical leaching has been partly replaced with bioleaching, i.e., with the industrial application of acid-producing leaching microorganisms. The microbiological mining of valuable and industrially important metals currently accounts for a significant part of the world metal production. The use of microorganisms makes it possible to extract valuable and significant metals with an initial content of less than 1%, which leads to a more rational and complete use of natural and secondary resources.

The most commonly used types of leaching microorganisms are acidophilic autotrophic bacteria or archaea which oxidize ferrous iron to ferric iron and reduced sulfur compounds to sulfuric acid. The use of acidophilic microorganisms that form acids, including sulfuric acid, causes less damage to the environment than the use of strong chemical acids in mining and recovery metals from wastes [4]. A separate little-studied area is the use of neutrophilic or moderately acidophilic microorganisms, leading to leaching due to the formation of organic acids.

Uncontrolled formation of acids by microorganisms can lead to corrosion of concrete, cement, and metal structures. Biocorrosion processes and their control present a serious problem for modern technologies and building operation. To solve these problems, microbiologists and biogeotechnologists should be involved [5]. The acidification of mining areas also requires the development of environmental remediation technologies and related problems of biocorrosion. The development of biogeotechnologies for mining is impossible without studying the diversity of microorganisms that carry out the leaching. Along with this, the development of methods to counteract biocorrosion of mineral materials also requires studying the diversity of microorganisms involved in these processes. Biomining and biocorrosion represent the positive and negative aspects of microbial leaching. Due to their practical significance, microbiological studies in these areas are inextricably linked with experimental modeling.

It should be noted that applied microbiology of the environment has not only great technological significance but also contains a number of topics, the solution of which is interesting for expanding basic microbiological knowledge on the whole. Examples of such areas can be the study on non-traditional energy sources for microorganisms in bioleaching and bioremediation, such as

- Phosphate removal via anaerobic bacterial phosphate reduction to phosphine [6,7];
- Aerobic bacterial oxidation of antimony [8,9];
- Bacterial utilization of by-products of rocket fuel [10,11];
- Use of organic C_1_ compounds by autotrophic bacteria [12,13].

The journal *Microorganisms* has already dedicated focus to applied environmental microbiology for years. Relevant publications in recent years include the Special Issue “Microorganisms for Environmental and Industrial Applications” (2017) and separate articles in various issues of the journal. Overall, the recent publications of the journal *Microorganisms* included, for example, themes of stimulation of neutrophilic autotrophic leaching bacteria [13] and the bioleaching of gold from ore concentrates and electronic waste [14]. The current specific issue and its reports will help to improve our understanding of modern applied environmental microbiology and the unity of its three constituent parts, namely biogeotechnology, biocorrosion, and remediation.
